# Experimental and Numerical Investigation of Tensile and Flexural Behavior of Nanoclay Wood-Plastic Composite

**DOI:** 10.3390/ma14112773

**Published:** 2021-05-24

**Authors:** Mohammad E. Golmakani, Tomasz Wiczenbach, Mohammad Malikan, Seyed M. Mahoori, Victor A. Eremeyev

**Affiliations:** 1 Department of Mechanical Engineering, Mashhad Branch, Islamic Azad University, Mashhad 9187144123, Iran; m.e.golmakani@mshdiau.ac.ir (M.E.G.); me.mahvari@gmail.com (S.M.M.); 2 Department of Mechanics of Materials and Structures, Gdansk University of Technology, 80-233 Gdansk, Poland; mohammad.malikan@pg.edu.pl (M.M.); victor.eremeev@pg.edu.pl (V.A.E.); 3 Department of Civil and Environmental Engineering and Architecture, Università degli Studi di Cagliari, Via Marengo, 2, 09123 Cagliari, Italy

**Keywords:** wood-plastic composite, nanoclay, polyethylene, wood powder, mechanical properties

## Abstract

In this study, the effect of wood powder and nanoclay particle content on composites’ mechanical behavior made with polyethylene matrix has been investigated. The wood flour as a reinforcer made of wood powder was at levels of 30, 40, and 50 wt.%, and additional reinforcement with nanoclay at 0, 1, 3, and 5 wt.%. Furthermore, to make a composite matrix, high-density polyethylene was used at levels of 70, 60, and 50% by weight. Wood-plastic composite (WPC) specimens were manufactured in injection molding. After preparing the specimens, tensile and bending tests were performed on samples. The mechanical properties such as tensile and flexural strength and flexural modulus were measured. Results showed that nanoclay particle content increases flexural modulus, flexural strength, modulus of elasticity, and tensile strength. The experimental test results show that Young’s moduli increased with the volume of wood flour. The biggest modulus of elasticity was achieved in the samples having 50 wt.% of wood powder. Furthermore, the highest value of tensile strength was achieved at the level of 30 wt.%. The highest flexural strength was for the sample containing 50% wood powder by weight. Additionally, a numerical model was made utilizing the Abaqus software using the finite element method (FEM). Comparing the numerical and experimental results, it was found that they are compatible in the linear-elastic and plastic state of the material. There are no crucial differences between experiment and FEM.

## 1. Introduction

Wood-plastic composite (WPC) is a promising and sustainable green material with similar properties to wood but with better water resistance and characterized by satisfactory stability of properties in many environments [1,2]. In the WPCs, the filler comprises waste wood in various forms, such as chips, sawdust, shavings, splinters, or the most common, wood flour. Due to the unique combination of polymer and wood properties and their attractive appearance, WPCs have become an interesting alternative for both polymers and wood industry products [3]. Their main advantage is using the same processing tools as their natural counterpart, wood [4].

According to the thermoplastic matrix, these composites can be used where natural wood does not meet the conditions necessary for its operation due to its resistance to water and biological corrosion [5,6]. Simultaneously, using a thermoplastic matrix allows them to be processed with classical polymer processing methods, which has a beneficial effect on production. Polyethene (PE), polypropylene (PP), and polyvinyl chloride (PVC) are usually used as the WPC matrix.

Another significant advantage of WPCs is that they have unique properties derived from combining the beneficial characteristics of wood and thermoplastic. Polymer composites filled with wood flour present favorable mechanical properties and higher stiffness compared to unfilled polymer materials [7]. Furthermore, compared with wood and wood-based materials, they are characterized by much lower water absorption and resistance to external (e.g., atmospheric) factors [8]. Moreover, applying waste wood materials as fillers for WPC is very favorable for ecological reasons. They are not a hazard to the natural environment during processing and recycling processes. Additionally, after proper modification, WPCs become partially biodegradable [9,10].

Moreover, the improvement of WPCs’ development can be achieved with nanoscience and nanotechnology. The use of nanosized fillers in WPC production can improve their mechanical and physical properties [11]. Depending on the expected properties, the types of nanofillers can be chosen. The most popular nanoclays are obtained from layered silicates. This nanosized filler usage can improve flexural and tensile strength and decrease water absorbance [12]. These properties can be significantly enhanced by increasing the interfacial area of polymer and wood flour. The particle size of nanoclays is less than 2 microns.

What is more, a small quantity of nanoclays can increase the mechanical properties of the composite. Nanoclays, with their high aspect ratio and nanometer size, provide increased particle–particle and polymer–particle interactions compared to traditional fillers [13]. Additionally, the addition of nanofiller in the composite decreases thickness swelling and water absorption.

Polymer nanocomposites provide additional properties with low filling (3–5 wt.% on average, max. 10 wt.%). This allows maintaining all the advantages of the original composite simultaneously. The essential benefits of nanoparticles in WPC production are increased stiffness without loss of impact strength, dimensional stability, improvement of the barrier effect, and reduced surface defects of products [14].

Sharma et al. [15] conducted a polymer-based composite with glass fiber and nanoclay reinforcements. They found that by increasing the amount of nanoclay up to 3%, tensile strength increased, and by adding nanoclay more than 3%, the strength decreased.

Islam et al. [16] reinforced the epoxy matrix with carbon nanotubes and nanoclay. After preparing the samples, a bending test was performed. The results of this experiment showed that the flexural strength is improved by adding nanoparticles. According to the impact test, it could be concluded that the samples reinforced with nanoparticles can absorb more energy than the control samples.

In a study, Deepak et al. [17] investigated the effect of using coconut shell and nanoclay as a reinforcing material in polyester. The results show that by increasing nanoclay, tensile strength, impact energy, and compressive strength increase.

Zunjarrao et al. [18] investigated the effect of nano- and micro-sized aluminum particles as reinforcements in the epoxy matrix. Failure test for three different samples of pure epoxy, particle-reinforced epoxy 20–100 μm, and epoxy reinforced with particles of 3–4.5 μm were conducted. Observations show that with the addition of particles to the epoxy, the toughness increases. The toughness of epoxy reinforced with particles of 20–100 μm is higher than that of epoxy with particles of 3–4.5 μm. Failure occurs at higher loads in both specimens, but this improvement is more significant for 3–4.5 μm particles than 20–100 μm.

In a study conducted by Srinivasa et al. [19], arka fruit peel was placed in the epoxy matrix as a reinforcing material. An impact test was performed on the specimens. Each sample was tested five times, and the average of the five tests was considered. The results of this experiment show that the strength of composites increases with increasing component volume.

Plastics have low strength due to their low Young’s modulus, but their applications are very wide. The main advantages of plastic materials are their low weight and applicability for high humidity environment. On the other hand, wood powder as reinforcement is a relatively cheap material. Its use is very extensive, as shown in the cited articles. Creating a composite from these two materials gives an excellent application in various engineering fields. Unfortunately, like each of the composite materials, its physical and mechanical properties are varied in the context of the different content of individual materials. Additionally, one of the newest solutions to strengthen materials is nanoparticles, e.g., nanoclay. The additional reinforcement resulting from the different content of these molecules strengthens the final material to some point. Therefore, the main objective of this paper is to investigate the mechanical properties of WPC containing different amounts of wood powder and strengthened with nanoparticles. Additionally, numerical analysis was performed to evaluate the experimental results. What is more, the mechanical properties of WPC containing different amounts of wood powder are given. Moreover, this research could influence the design of WPC products, considering the increase of mechanical and physical properties.

## 2. Materials and Methods

### 2.1. Materials

In this research, the matrix material is heavy polyethylene prepared from Arak Petrochemical Trading Company (Tehran, Iran) with code 5218 and a melt flow index of 18 g/10 min, the specifications of which are given in Table 1.

The nanoclay used for the preparation of the specimens was prepared by Sigma-Aldrich company (Munich, Germany). The characteristics of the nanoclay are summarized in Table 2. Narad wood flour was used as a wood powder in the polymer matrix. Kian Choob Company (Tehran, Iran) prepared the wood flour. The wood flour was classified using a vibrating sieve to uniform the particle size and reach the desired size. Firwood flour, which passed through 60 mesh, and the rest on a sieve with 80 mesh, was considered flour. The samples were then placed in an oven at 100 °C for 24 h to dry.

The coupling agent maleic anhydride was used. Its specifications are presented in Table 3.

### 2.2. Preparation of Wood-Plastic Composite

The mixing process was performed using the internal mixer HBI System 90 made by the American company Haake Buchler (New Jersey, NJ, USA). A mixing temperature of 170 °C and mixing speed of 60 RPM were used. The total mixing time until reaching constant torque was 10 min. First, polyethylene was poured into the device’s housing, and after melting for 2 min and reaching constant torque, maleic and nanoclay were added. After 5 min, flour was added. The wood was poured into the chamber, and the mixing operation was carried out continuously until a constant torque was reached.

After cooling and hardening, the material was granulated using the Wieser WG-Ls 200 semi-industrial shredder (Wieser Company, Hamburg, Germany). The compounds of each specimen are shown in Table 4.

**Figure 1:**
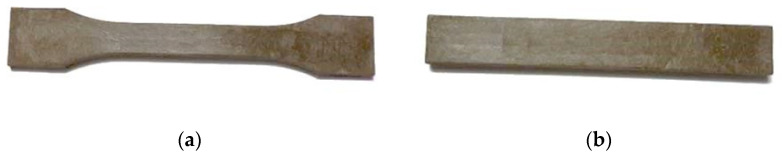
Specimens for: (**a**) tensile test; (**b**) bending test.

**Figure 2:**
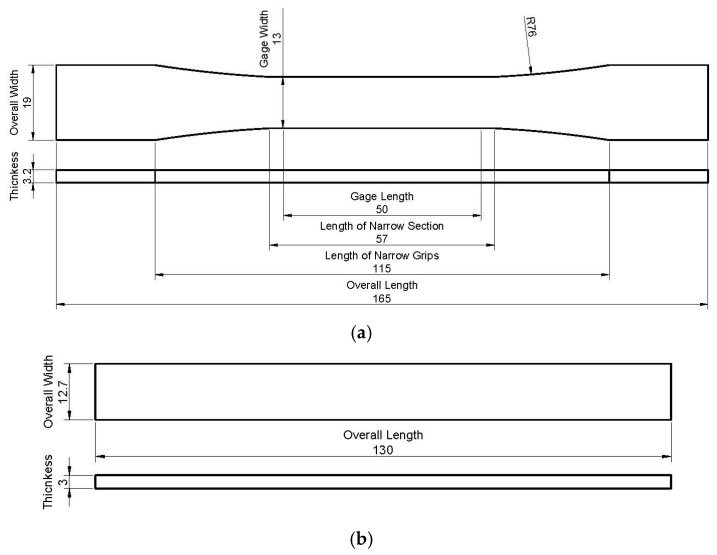
Dimension of samples [mm] for (**a**) tensile test; (**b**) bending test.

The granules were then transferred to an injection molding machine to make standard test specimens. For this purpose, a semi-industrial injection machine made by Imen Machine Company (Tehran, Iran) of Tehran was used. The injection cylinder temperature in each region was 155, 165 and 175 °C. The injection pressure was 100 MPa and lasted for less than 75 s. The mold was cooled with cold water, and the samples were taken out of the mold after 2 min. The injection mold was designed so that the specimen, according to ASTM D638-02A [20] and ASTM D790-02 [21] standards, for tensile, bending, and impact tests were obtained. Figure 1 shows a sample taken out of the mold. Figure 1a presents the specimen for the tensile test, and Figure 1b shows the bending test sample. Additionally, Figure 2a,b show the dimensions of samples for the tensile and bending test, respectively.

### 2.3. Mechanical Testing

To evaluate the mechanical properties of wood-plastic composite, the tensile and bending test, according to ASTM D638-02A and ASTM D790-02, respectively, were performed. The experimental tests of specimens were done on the testing device Instron 4486 at room temperature. All tensile and bending tests were conducted applying a 500 N load cell. What is more, the crosshead speed moved at 2 mm/min. All performed mechanical testing of each sample composition was performed no fewer than five times, and the mean values were computed and taken into consideration. According to obtained force-displacement curves, the calculations were performed to obtain tensile strength, flexural strength, and Young’s moduli. Additionally, to calculate the Poisson’s ratios, the two-way TML strain gauge was installed on the test specimens. Measurement with this device allowed to the calculation of the Poisson’s ratios.

### 2.4. Statistical Analysis

In this study, SPSS 24 software (24.0, IBM Corp., Armonk, NY, USA) was used. Statistical analysis of data was performed using variance analysis and comparison of mean values using Duncan’s test. The variable factor’s effect on the studied properties was investigated at a 95% confidence level (5% significance level).

### 2.5. Numerical Analysis

#### 2.5.1. Tensile Test Simulation

The WPC specimen was modeled following the shape and dimensions of the ASTM D638-02A standard in Abaqus software version 6.14.1 (Dassault Systemes, ABAQUS Inc., Waltham, MA, USA).

The properties of two-phase elastic and plastic materials for the wood-plastic composite were considered. In the linear-elastic region, the material was deemed to be isotropic. The Young’s modulus and Poisson’s coefficient were entered for each sample’s according to Table 5. Then plastic behavior was modeled. First, the yield stress and plastic strain zero value were entered as the plastic strain corresponding to the yield stress to define the plastic region’s material properties. Then, in the next row, the stress greater than the yield stress and the corresponding plastic strain was entered.

The boundary conditions of one side of the specimen at the device’s fixed grip was motionless, and the other side could move in a neutral axis direction. Mesh size was selected as 1 mm. The model was prepared only for half of the specimen because of symmetry. Finally, the sample was aligned with the hexagonal elements in a regular and uniform form. Then, the analysis was performed with Abaqus software.

**Figure 3:**
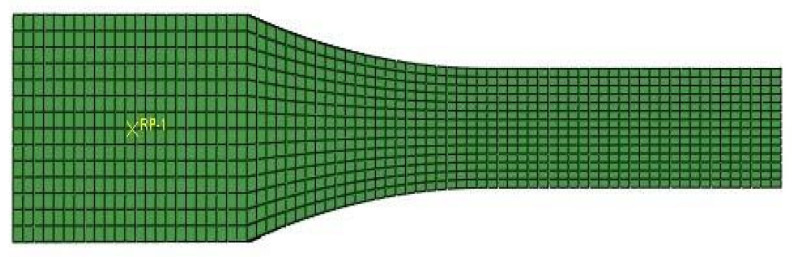
Specimen model for the numerical tensile test.

Figure 3 shows the meshed specimen for the numerical tensile test.

#### 2.5.2. Bending Test Simulation

The WPC specimen was modeled following the shape and dimensions of ASTM D790-02 standard in Abaqus software version 6.14.1. The mechanical properties of each specimen are considered, according to Table 5. One of the grips was deemed immobile for the boundary conditions, and the second grip was movable only vertically. Then, two supports and a load-applying device, which are cylindrical, were modeled. The contact of the upper grip with the sample surface was defined in the interaction module with the surface-to-surface mode. Then, using a rigid body constraint, the lower supporting grips and the upper movable grip were modeled as rigid, and their movement was limited to the movement of a point. In the loading module and in the boundary condition section, the lower supporting grips, whose movement was limited to one point, were considered immobile. The numerical value of the upper movable grip displacement was entered. The amount of movable grip displacement is obtained from the experimental test. The mesh size of 1 mm was finally selected. The hexagonal elements in regular and uniformed form were assigned.

**Figure 4:**
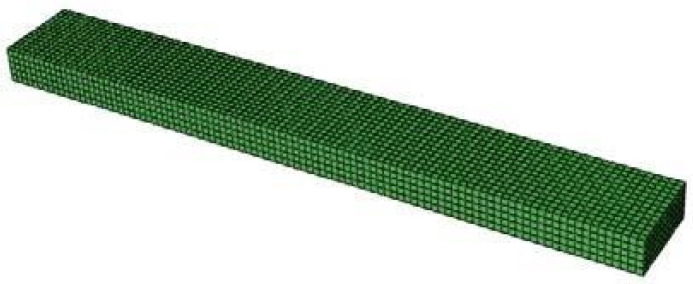
Model of the specimen for the numerical bending test.

Figure 4 shows a modeled specimen for a bending test.

## 3. Results

### 3.1. Experimental Analysis

Stress–strain curves are generally obtained through tensile tests, which are limited by the phenomenon of choking. Engineering stress–strain curves are converted to real stress–strain curves using direct relationships. The stress and strain values obtained from the tensile test were converted to real stress and strain values using the following formula. The amount of stress obtained from the tensile test using Equation (1) and the strain obtained from the tensile test using Equation (2) were converted into real stress and strain values. What is more, the amount of plastic strain was obtained using Equation (3).

(1) $$\sigma_{\mathrm{True}}=\sigma_{\mathrm{Num}}\left(1+\varepsilon_{\mathrm{Num}}\right),$$

(2) $$\varepsilon_{\mathrm{True}}=\ln\left(1+\varepsilon_{\mathrm{Num}}\right),$$

(3) $$\varepsilon_{P}=\varepsilon_{\mathrm{True}}-\frac{\sigma_{\mathrm{True}}}{E},$$

where $\sigma_{\mathrm{True}}$ is the real stress, $\varepsilon_{\mathrm{True}}$ is the real strain, $\varepsilon_{\mathrm{Num}}$, is the engineering strain (from the tensile test), and $\varepsilon_{P}$ is the plastic strain.

The values of Young’s moduli, Poisson’s ratio, stress and strain are taken from the experimental tensile test and are shown in Table 5.

The addition of nanoclay to the samples showed that the modulus of elasticity in all three levels of 30%, 40% and 50% wood, with 5% nanoclay to the samples compared to other values, had the highest value. Table 6 shows the modulus changes corresponding to the changes in wood powder and nanoclay. Examining the variance analysis in the modulus of elasticity showed that changes in nanoclay at levels of 0%, 1%, 3% and 5% had a significant effect on the statistical confidence level 95%. According to Duncan grouping, two different groups were examined. The first one is with 0% and 1% nanoclay and the second with 3% and 5% values. The highest modulus was obtained in the second group, according to this test.

The sample with 1% nanoclay had the highest resistance. Examining the tensile strength variance showed that changes in the amount of nanoclay at levels of 0%, 1%, 3% and 5% have no significant effect. Table 7 presents the results of nanoclay on tensile strength.

The effect of adding nanoclay to the samples showed that at the level of 30% and 40% wood flour, the specimen containing 3% nanoclay had the highest flexural modulus. Still, at the 50% wood flour level, the sample containing 1% nanoclay had the highest flexural modulus. Table 8 shows the amount of nanoclay impact on the flexural modulus.

Table 9 shows the amount of nanoclay impact on flexural strength. Examining the variance analysis in the flexural modulus showed that changes in the amount of nanoclay at levels of 0%, 1%, 3% and 5% significantly affected the statistical confidence level of 95%. Duncan grouping for the impact of nanoclay on the flexural strength divided the four specimens into two separate groups, so specimens of 0% and 3% are in the first group with the lowest flexural strength. Values of 1% and 5% nanoclay are in the second group with the highest flexural strength value. The effect of adding nanoclay to the samples showed that at the level of 30% wood flour, the model containing 3% nanoclay had the highest flexural strength. Still, at the levels of 40% and 50% wood flour, the specimen with 5% nanoclay has the highest flexural strength.

**Figure 5:**
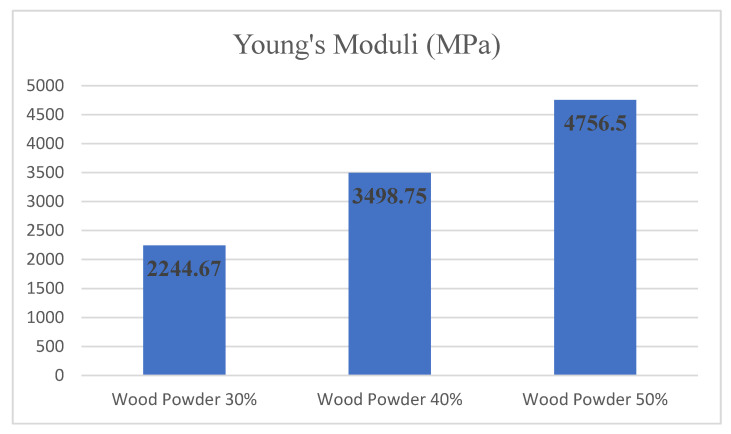
The Young’s moduli according to the amount of wood powder and 0% nanoclay addition.

Figure 5 shows the modulus changes based on the amount of wood powder for the samples containing 0% nanoparticles. Examination of the tensile test results shows that in the sample containing 50% wood particles, the modulus of elasticity is 4756.50 MPa, the highest obtained elastic modulus. The specimens containing 30% and 40% wood particles had Young’s moduli which reached 2244.67 and 3498.75 MPa, respectively. As it can be seen, by having 50% wood powder compared with 30% wood powder content, Young’s moduli doubled its value.

**Figure 6:**
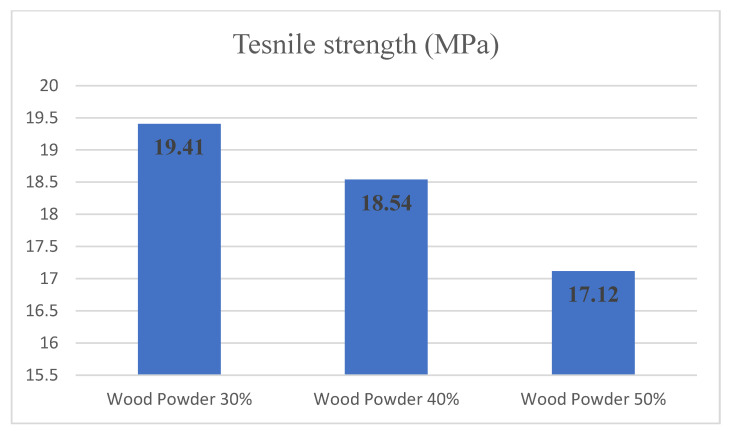
Tensile strength comparison according to a different amount of wood powder and 0% nanoclay.

Figure 6 presents the tensile strength changes following the amount of wood powder for the specimens with 0% nanoclay. The tensile test results have shown that in the sample containing 30% wood particles, the tensile strength was equal to 19.40 MPa. By changing the amount of wood particles to 40 and 50%, the value of tensile strength reached 18.54 and 17.12 MPa, respectively. According to the tensile test values, wood particles in all three levels have reduced the tensile strength.

**Figure 7:**
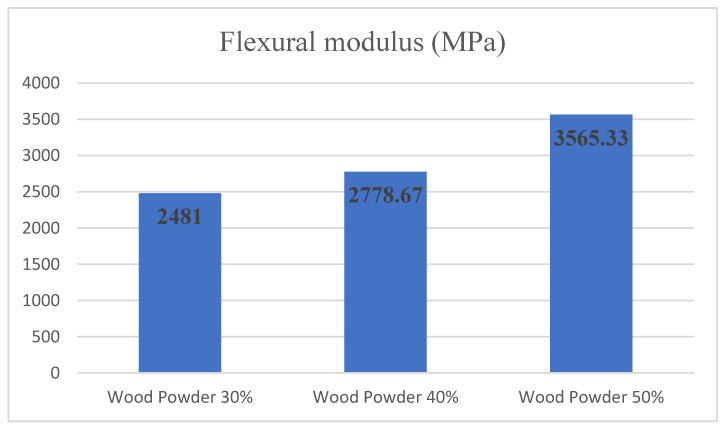
Flexural modulus comparison according to a different amount of wood powder and 0% nanoclay.

Figure 7 shows the changes in the modulus of elasticity relative to the amount of wood flour. Examining the bending test results shows that the addition of wood particles has increased the flexural modulus in all three levels. Examining the flexural modulus variance analysis showed that changes in the amount of wood at levels of 30%, 40% and 50% significantly affected the statistical confidence level of 95%. Duncan grouping also placed each level of wood values in a separate group to impact the amount of wood on flexural modulus. Hence, samples with 50% wood powder had the highest value of the flexural modulus.

**Figure 8:**
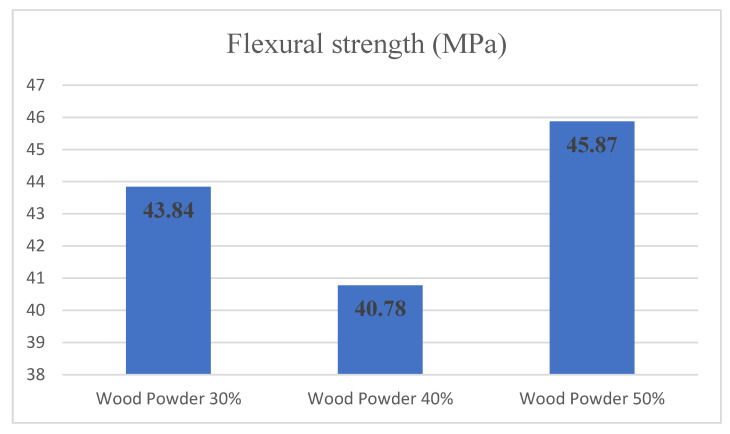
Flexural strength comparison according to a different amount of wood powder and 0% nanoclay.

Figure 8 shows the changes in flexural strength relative to changes in the amount of wood flour. Examining the variance analysis in flexural strength showed that changes in the amount of wood at levels of 30%, 40% and 50% significantly affected the level of statistical confidence of 95%. Duncan grouping also divided the three levels of wood flour into two separate groups to assess the wood particles’ impact on flexural strength. The specimens containing 30% and 40% by weight of wood particles are in the first group with the least amount of flexural strength, and the 50% by weight wood particles are in the second group with the highest amount of flexural strength.

### 3.2. Numerical Analysis

The failure tensile and bending force values for both the laboratory and numerical test are shown in Table 10 and Table 11 with the percentage difference, respectively. As shown in the table, there is a difference between the experimental and the numerical results.

## 4. Discussion

The Young’s moduli increase in nanofiller composites is directly related to the average length of clay particles and their dimensional ratio. Various structural factors such as volume ratio, nanoclay aspect ratio, particle spacing, and the amount of nanofiller particle cracking have a significant effect on the mechanical properties of clay-polymer composites. Additionally, the difference between the degree of swelling of the layers and the layer structure formation strongly affects the nanoclay composite’s elasticity modulus. Besides, the percentage of nanofiller in composites plays a significant role, usually increasing the mechanical properties with the percentage of nanoclay [22]. However, after a specific limit, the increasing trend of properties stops or even decreases with the increment of the nanofiller particles [23].

Increasing the nanoclay, interlayer, and layer morphology in the nanofiller composite, due to the interfacial effect of organic chains and nanoclay particles and the orientation of layered silicate particles, increases the strength of the nanofiller composite. Additionally, the heterogeneity and high surface-to-volume ratio of nanoclay with organic matter contribute to the high-reinforcing capacity of nanofiller particles. It is done so that nanoparticles as a reinforcer causes the joint surface between two phases. As a result of swelling of the clay layers and strong adhesion between the polymer and the clay, the composite increases in strength [24]. Therefore, nanoparticles increase the tensile and flexural strength of WPC [25].

The coupling agent’s percentage increases the joint strength between the two phases of cellulosic and polymer. In other words, the use of a coupling agent creates a more homogeneous structure in the composite. It causes a better stress distribution during the application of static load. Less stress concentration will occur in one area of the material, increasing stress-bearing capacity and flexural strength. To effectively transfer the stress and distribute the load correctly, it must have a strong connection at the two phases’ joint surface. Without a coupling agent, nanofiller particles are present as separate components with weak bonds within the substrate. Therefore, they cannot participate effectively in the stress distribution [26,27].

Since the amount of wood particles added increases the modulus of elasticity in all three levels, and as lignocellulosic materials have a higher modulus of elasticity, so the value of Young’s moduli of the composite material increases with the addition of wood flour to polyethylene [28]. The modulus increase indicates a decrease in the composite material’s deformation under load, which is a positive factor in engineering structures that must withstand a large load without deformation [29]. Therefore, increasing the modulus of elasticity concerning the relationship E = σ/ɛ and decreasing the samples’ strain by increasing the amount of wood flour can also be justified.

In WPC, with high numbers of fibers, the matrix has the role of adhesive for bonding wood particles to each other [30]. This leads to a mechanical bond between the wood and polymer particles. Lack of chemical bonding is due to plastic’s non-polar nature and wood fibers’ polar nature [31]. The volume of wood powder in the polyethylene leads to the polymer’s cracking and reduces strain. On the other hand, with more wood flour, the strain values have been significantly reduced. Therefore, according to the relation Eɛ = σ, the value of the modulus of elasticity increased, causing a sharp decrease in the value of strain and reduced tensile strength.

Furthermore, the mechanical test was simulated using Abaqus software. The results indicate a difference between the failure force’s values from the experimental test and numerical analysis. This difference may be due to the test sample’s adjustment in the device’s grips. This situation is minimized when closing the specimens, but it does not disappear completely. However, in software simulation, the problem is entirely ideal. Furthermore, in theory, and numerical simulation, the model is created as a continuous medium. In other words, the whole body is filled with material particles. In the experimental part, voids filled with oxygen may arise during the molding process, providing different results, such as fracture force. What is more, when the WPCs matrix exceeds its maximum capacity, cracks will appear. This effect will decrease the stiffness of the whole material and contribute to the difference between experimental and numerical results. More importantly, as we used fixtures to do the mechanical tests, some loss between jaws and the part existed. However, the part modeled in the FEM software is entirely fixed, and this discrepancy can result in some differences in the numerical results of experimental vis-à-vis FEM.

## 5. Conclusions

In summary, the mechanical properties of WPCs reinforced with the addition of nanoparticles are investigated. As reported in this manuscript, the inclusion of nanoclay particles has a significant effect on the material’s physical and mechanical properties. The heterogeneity and high surface-to-volume ratio of nanoclay with organic matter contribute to nanoparticles’ enhancement capability. It is done so that the nanoclay particles, as reinforcements, increase the surface area. On the other hand, with the addition of clay, the composite’s strength increases due to swelling of clay layers and strong surface adhesion between the polymer and clay. For this reason, the results show that with increasing the amount of nanoclay and wood powder, the tensile and flexural strength of WPC has increased [32].

- Since nanoclay particles create stress areas and cracking points of failure, as nanoclay increases, the impact resistance of the composite decreases.
- The presence of nanoclay increases the energy absorbed by the composite, so increasing the amount of nanoclay creates areas in the polymer matrix that cause more stress, and cracking starts from that area [32].
- The results show that by increasing the amount of nanoclay, the impact resistance of the WPC specimens has decreased [33].
- The increase of Young’s modulus and flexular modulus in the composite consisting of nanoclay depends directly on the average length of nanoclay particles and their dimensional ratio.
- The difference between the amount of shrinkage with increasing Young’s modulus and flexular modulus of WPC has increased [34,35].

The results of mechanical tests showed that the amount of wood powder impacts Young’s modulus.

- This phenomenon is due to lignocellulosic materials having higher Young’s moduli than polymer. So the value of the modulus of elasticity of the composite material increases with the addition of wood flour to polyethylene [28,29].
- With the addition of wood powder, the strain values reduce. Accordingly, the relation σ = Eɛ, with the increased Young’s moduli and decreased strain value reduction of tensile strength is obtained [30,31].

## Figures and Tables

**Table 1:** Specifications of polyethylene.

Melt Flow Index after Recycling (g/10 min)	Melt Flow Index (g/10 min)	Density (gr/cm^3^)
35.04	18	0.956

**Table 2:** Specifications of polyethylene bonded with maleic anhydride.

Properties	K10
Organic Modifier	MT2EtOH
Base	Montmorillonite
Density	300–370 kg/m^3^
Anion	Chloride
Modifier Concentration	48 meq/100 g
Moisture	1–2%
Weight Loss on Ignition	30%
According to Producer Information	

**Table 3:** Specifications of polyethylene bonded with maleic anhydride.

The Amount of Anhydride Bonded (wt.%)	Melt Flow Index (g/10 min)	Density (gr/cm^3^)
1%	7	0.965

**Table 4:** The number of compounds in each specimen.

Specimen	PE (%)	MA (%)	Nanoclay (%)	Wood Flour (%)
1	70%	3%	0%	30%
2	70%	3%	1%	30%
3	70%	3%	3%	30%
4	70%	3%	5%	30%
5	60%	3%	0%	40%
6	60%	3%	1%	40%
7	60%	3%	3%	40%
8	60%	3%	5%	40%
9	50%	3%	0%	50%
10	50%	3%	1%	50%
11	50%	3%	3%	50%
12	50%	3%	5%	50%

**Table 5:** Material properties for each specimen.

Specimen	σ_y_ (MPa)	σ_num_ (MPa)	ε_p_ (-)	E (MPa)	ν (-)
1	14.61	19.41	0.0243	2244.67	0.3422
2	14.68	19.11	0.0253	2314.00	0.3988
3	14.20	16.80	0.0165	2325.25	0.4435
4	13.86	18.98	0.0157	2355.75	0.4800
5	13.83	18.54	0.0088	3498.75	0.3520
6	13.70	18.31	0.0081	3307.25	0.4052
7	14.10	20.34	0.0111	4130.33	0.4549
8	14.20	20.52	0.0119	4184.00	0.4883
9	14.75	17.12	0.0037	4756.50	0.3750
10	15.88	20.52	0.0053	4899.67	0.4285
11	12.80	18.14	0.0047	4951.25	0.4684
12	13.12	19.59	0.0045	5426.25	0.4955

**Table 6:** The modulus of elasticity according to the changes in wood flour and nanoclay.

Specimen	Wood Flour (%)	Nanoclay (%)	Young’s Moduli (MPa)
1	30%	0%	2244.67
2	30%	1%	2314.00
3	30%	3%	2325.25
4	30%	5%	2355.75
5	40%	0%	3498.75
6	40%	1%	3304.25
7	40%	3%	4130.33
8	40%	5%	4184.00
9	50%	0%	4756.50
10	50%	1%	4899.67
11	50%	3%	4951.25
12	50%	5%	5426.25

**Table 7:** Impact of nanoclay on tensile strength.

Specimen	Wood Flour (%)	Nanoclay (%)	Tensile Strength (MPa)
1	30%	0%	19.41
2	30%	1%	19.11
3	30%	3%	16.80
4	30%	5%	18.98
5	40%	0%	18.54
6	40%	1%	18.31
7	40%	3%	20.34
8	40%	5%	20.52
9	50%	0%	17.12
10	50%	1%	20.52
11	50%	3%	18.14
12	50%	5%	19.59

**Table 8:** The flexural modulus according to the amount of nanoclay.

Specimen	Wood Flour (%)	Nanoclay (%)	Flexural Modulus (MPa)
1	30%	0%	2481.00
2	30%	1%	2372.67
3	30%	3%	2610.67
4	30%	5%	2602.33
5	40%	0%	2778.67
6	40%	1%	2946.00
7	40%	3%	3320.67
8	40%	5%	3210.33
9	50%	0%	3565.33
10	50%	1%	4186.67
11	50%	3%	3452.67
12	50%	5%	3905.67

**Table 9:** The flexural strength according to the amount of nanoclay.

Specimen	Wood Flour (%)	Nanoclay (%)	Flexural Strength (MPa)
1	30%	0%	43.84
2	30%	1%	41.48
3	30%	3%	46.17
4	30%	5%	45.72
5	40%	0%	40.78
6	40%	1%	44.75
7	40%	3%	46.84
8	40%	5%	47.18
9	50%	0%	45.87
10	50%	1%	48.41
11	50%	3%	46.84
12	50%	5%	50.82

**Table 10:** Comparison of failure force obtained in the experimental and numerical tensile test.

Specimen	Wood Flour (%)	Nanoclay (%)	Experimental (kN)	Numerical (kN)	Difference (%)
1	30%	0%	0.7762	0.8523	9.80%
2	30%	1%	0.7644	0.7979	4.30%
3	30%	3%	0.7181	0.7799	8.60%
4	30%	5%	0.7751	0.7717	0.40%
5	40%	0%	0.7385	0.8093	9.60%
6	40%	1%	0.7239	0.7887	8.90%
7	40%	3%	0.8136	0.8651	6.30%
8	40%	5%	0.8155	0.8675	6.40%
9	50%	0%	0.6593	0.7630	15.7%
10	50%	1%	0.8208	0.8504	3.60%
11	50%	3%	0.6782	0.7075	4.30%
12	50%	5%	0.7993	0.7697	3.70%

**Table 11:** Comparison of the failure force in the experimental and numerical bending test.

Specimen	Wood Flour (%)	Nanoclay (%)	Experimental (kN)	Numerical (kN)	Difference (%)
1	30%	0%	0.1054	0.0941	12.01%
2	30%	1%	0.1022	0.0882	15.87%
3	30%	3%	0.1069	0.0867	23.30%
4	30%	5%	0.1078	0.0839	28.49%
5	40%	0%	0.1002	0.0879	13.99%
6	40%	1%	0.1045	0.0885	18.08%
7	40%	3%	0.1116	0.0952	17.23%
8	40%	5%	0.1143	0.0949	20.44%
9	50%	0%	0.1129	0.0878	28.59%
10	50%	1%	0.1124	0.0877	28.16%
11	50%	3%	0.1155	0.0885	30.51%
12	50%	5%	0.1196	0.0958	24.84%

## Data Availability

Data available on request due to restrictions e.g., privacy or ethical. The data presented in this study are available on request from the corresponding author. The data are not publicly available due to privacy of results belonging to the Islamic Azad University.
